# Leveraging health financing, digital health and self-care approaches to strengthen maternal health journeys in India: perspectives from Assam

**DOI:** 10.3389/fgwh.2025.1469328

**Published:** 2025-04-09

**Authors:** Sowmya Ramesh, Charlotte E. Warren, Ben Bellows, Himanshi Dwivedi, Himani Gupta, Ashita Munjral, Swapnil Rawat, David Tresner-Kirsch, Jitender Nagpal

**Affiliations:** ^1^ Population Council Consulting, New Delhi, Delhi, India; ^2^ Social and Behavioral Science Research, Population Council, New York, NY, United States; ^3^ Nivi Inc, Sudbury, MA, United States; ^4^ Population Council, New Delhi, Delhi, India; ^5^ Research Department, Sitaram Bhartia Institute of Science and Research, New Delhi, Delhi, India; ^6^ Department of Pediatrics, Sitaram Bhartia Institute of Science and Research, New Delhi, Delhi, India

**Keywords:** maternal health, health finance, chatbot, digital support system, empowerment, selfcare, Assam, India

## Abstract

Maternal morbidity and mortality in India continue to be high in populations and places with limited access to quality health services. Major barriers include out of pocket expenditure, lack of autonomy and information around maternal health services and weak implementation of pro-poor policies. Addressing demand-side barriers and enablers is critical to improving healthcare uptake and healthcare adherence along the pregnancy-postnatal continuum. This paper describes three well known operational spaces, maternal health financing, digital health, and self-care interventions within the Indian context including pro-poor maternal health policies, mobile health ecosystems and networks, and self-care opportunities that promote women's knowledge, choice, self-efficacy, and autonomy. These are expanded on to identify additional opportunities to improve access to MH services. Finally, the authors describe a new digital health intervention using a chat-based digital support system that has the potential to reduce barriers that women face in seeking and receiving quality MH services in Assam and elsewhere. Future work on how to implement such a combined approach need to account for multiple contextual factors, including understanding the nature and success of national pro-poor MH policies in each state, how the public and private health systems function and interact, social determinants of health as well as engaging women in the process to improve maternal and newborn health outcomes.

## Introduction

In India, the maternal mortality ratio declined from 398 deaths per 100,000 live births in 1997 to 99 deaths per 100,000 live births in 2020 (1). However, maternal, and newborn morbidity and mortality continue to be high in populations and places with limited access to quality health services. Sixty three percent of the 23,800 maternal deaths in 2020 occurred in poorer states, particularly nine northeastern and central states, and among women aged 20–29 years (58%) (1, 2). Uptake of antenatal care (ANC) is correlated with caste, education level, household income, urban living, birth order, and pregnancy intentions (3).

Major barriers to uptake of care include lack of autonomy, access to maternal health (MH) information, and distance to health facilities and transport costs. Out-of-pocket expenditure (OOPE) remains high with the poor paying more of their income towards MH care (4, 5). Moreover, women are not aware of India's pro-poor MH policies and benefits, resulting in poor early and continued ANC attendance or postnatal contacts (6) including limited opportunities for self-care.

Addressing demand-side barriers and enablers is critical to improving healthcare uptake and healthcare adherence along the pregnancy-postnatal continuum. Financial (pro-poor), digital health and self-care interventions are three well-known operational spaces with potential to address barriers that women face in seeking and receiving quality MH services. Interventions from these operational spaces are described within the MH context in India and expanded on to identify additional opportunities to improve access to MH services.

### Pro-poor maternal and newborn healthcare programs in India

There are a range of financial incentives within MH policy interventions to increase access to services and reduce OOPE (2). In response to high maternal and newborn morbidity and mortality, the Indian government introduced several pro-poor and quality improvement policies to improve women's access to quality MH care since 2005. The main pro-poor policies are described below:

1. ***Janani Suraksha Yojana* (JSY)** was launched in 2005 by the National Health Mission (NHM); a centrally sponsored scheme integrating cash assistance with delivery and post-delivery care. Pregnant women living below the poverty line receive a cash incentive if they give birth in an institution. JSY includes a payment to the community health worker (Accredited Social Health Activist– ASHA) who motivates and encourages women to have a facility-based birth and access ANC and PNC services. JSY appears to benefit women with no formal schooling and women living in rural areas (2). However, there is limited evidence that the program benefited the poorest women (7, 8) who had trouble proving their eligibility or did not have a bank account to receive payments (6, 9). While JSY reduced OOPE, with some success, families still suffer from catastrophic expenditure for obstetric complications (4). Nevertheless, after JSY women had more ANC contacts, longer stays after birth, breastfeeding within one hour of birth, and uptake of postnatal care (PNC) (10). The combination of ASHAs support and cash transfers (CT) among eligible women was higher than those women who only received a CT on the institutional birth rate and breast feeding initiation (11).
2. ***Janani Shishu Suraksha Karyakram (JSSK)*** was launched by The NHM in 2011 with the aim of reducing OOPE on MNH services. JSSK provides free MH services for all pregnant women in public health institutions, including caesarean section, diagnostics, blood, medicines, other consumables, food, and free return transport. There was a significant increase in ANC checkups and institutional delivery in public facilities due to JSSK, and since 2014, women are entitled to treatment for pregnancy and postnatal complications and sick babies up to one year of age—extending the potential for women to access care throughout their pregnancy -postnatal journey. However, many women reported paying OOPE on these services, and informal payments to staff. In Raipur, 89% of women incurred OOPE for institutional delivery. Public facilities lack staff for imaging or lab investigations and stockout of basic medicines, and women also report dissatisfaction concerning cleanliness and provider behaviour (12–14). More than 50% of pregnant women living in remote areas of Assam did not receive free ambulance services due to poor road conditions and, when available, use personal or hired vehicles to reach MH services instead (15).
3. ***Pradhan Mantri Surakshit Matritva Abhiyan (PMSMA****)* Launched in 2016 by the Ministry of Health and Family Welfare, PMSMA aimed to improve ANC uptake, especially in remote areas by providing a minimum package of investigations, including imaging, medicines, and nutritional supplements on a fixed day, every month. To mitigate staffing shortages in public facilities, private OB/GYN specialists, radiologists, and physicians volunteer their services for 12 days a year and ensure timely identification and referral of high-risk pregnancies (2). Despite ultrasonography being crucial to rule out high-risk pregnancies and PMSMA promising free scans, in one study, most women reported using private imaging laboratories for the ultrasounds (16).
4. ***Pradhan Mantri Matru Vandana Yojana* (PMMVY)** Launched in 2017 by the Ministry of Women and Child Development, to mitigate OOPE for ANC, delivery, and PNC; providing a CT to women who are employed and pregnant with or nursing their first child and additional incentives if a second child is a girl. Beneficiaries are also eligible for JSY. Some women report problems linking bank accounts and their Aadhar card slowing the CT disbursement (17). PMMVY guidelines state that women should receive funds within 30 days after registration, but there are backlogs in women receiving the CT.
5. ***Ayushman Bharat National Health Protection Mission (NHPM)*** launched in 2017 to move from a sectoral, segmented approach to a comprehensive needs-based healthcare service. It took on *Rastriya Swasthya Bima Yojana* (RSBY), which was originally launched in 2008 to advance universal health coverage as a national health insurance program for maternity-related hospitalization among “Below Poverty Line” households. In 2018 RSBY, which was implemented through insurance companies but subsidized by Union and State governments, was converted into Pradhan Mantri Jan Aarogya Yojana (PM-JAY) under NHPM*.* PM-JAY is a government-funded health insurance scheme that covers hospitalization costs in public and private hospitals for the poorest 40% of the population (around 500 million economically and socially disadvantaged Indians), providing up to US$7,000 per family per year. To enrol, a beneficiary must physically visit a health facility for identification and to receive a PM-JAY card. Beneficiaries are identified under the Socio-Economic Caste Census database for rural areas and 11 occupational criteria for urban areas, and in states where RSBY is still active (18).

### Digital interventions for improving maternal health in India

Over 80% of men in India and 71% of women own a mobile phone in India; 49% men and 26% women have smartphones although this varies between rural and urban settings (19). Nevertheless use of digital technology for health is an increasingly important priority for the Government of India (17). Digital platforms and mobile health applications that enable the delivery of services have huge further potential to improve access to care and health service delivery, despite low literacy levels (20–24). Most digital health interventions are phone-based and can engage individuals in their everyday lives, help them navigate into and across the health system, and source client-perspective feedback following client–provider interactions. Messaging systems using a digital application (“app”) or chatbot have shown a positive impact on improving attendance to 4 or more ANC contacts and facility delivery (25–27). The initial part of a chatbot MH journey involves discovering and onboarding the patient or pregnant woman into the chatbot, which can happen at a facility or in the community via a health care provider, an ASHA or the patient herself. Finding and enrolling women early in pregnancy into a chatbot before she visits the facility could improve her chances of having her first ANC contact in the first trimester. Most chatbots, at a minimum, provide gestation-appropriate information on the health of their baby, uptake of critical health promotive measures like tetanus toxoid injection and consumption of iron and folic supplements, as well as information about how to manage minor pregnancy side effects, pregnancy and childbirth danger signs, and reminders on ANC appointments (25, 27–30). These digital patient engagement systems may serve as a psychological support system for women and augment “perceived satisfaction of care” and improve their knowledge and health-seeking behaviour (27, 28).

The **Ayushman Bharat Digital Mission (ABDM**) was launched in 2021 to establish a national digital health ecosystem that is integrated, effective and inclusive, and aims to support the country's integrated digital health infrastructure through interoperable frameworks. “It bridges the existing gap amongst different healthcare ecosystem stakeholders through digital highways” (29). ABDM has several building blocks and includes registries of individual patients, a health provider registry, and a registry of health facilities, including hospitals, laboratories, and pharmacies.

**Kilkari,** an extensive maternal mobile messaging program, provided mobile health information to over 10 million subscribers in 2019. Kilkari delivers weekly, prerecorded audio information about reproductive, maternal, neonatal, and child health, starting in pregnancy (20 weeks) and continuing until the infant reaches 1 year (30, 31). Reports suggest that Kilkari has improved some maternal and child health behaviours, but subscriber retention is low, and it has not closed pre-existing gaps in indicators such as wealth and education (32, 33). As an intervention, it focuses on engaging mothers on health content. Geo-referenced referrals to facility and patient follow-up post-visit are not yet part of the platform, although likely to change in the future.

The next step in sharing reliable messaging periodically is to deploy chatbots. Chatbot deployment aims to improve health system navigation. Early models like the “askNivi” (34) chatbot can refer users to nearby healthcare providers. The necessary system integrations required to track user-initiated appointments are being developed and can be refined over time. For digital health initiatives like askNivi, which engage users and generate referrals to both public and private providers, validating the visits and catering to each user's post-visit needs will become more critical to prove as the platforms scale.

### Improving awareness and increasing opportunities for self-care and self-help groups in India

Health awareness interventions promote choice, self-efficacy, and autonomy. They can empower pregnant women with information, improving their awareness to make informed decisions on where and when to seek healthcare during pregnancy, childbirth, and childcare (32). This awareness can potentially increase women's self-care including decisions to access preventive services and take up preventive behaviours, improve treatment adherence, and reduce the need for formal healthcare services (35).

The WHO defines self-care as “the ability of individuals, families, and communities to promote health, prevent disease, maintain health and cope with illness and disability with or without the support of a health-care provider” (36, 37). Providing information on MH through self-help groups in India has been widely documented (38). Evidence suggests that self-help groups support women in following recommended health practices and improving health system access through community health workers (39). Participatory learning through these groups provides opportunities for women to engage with others, including fostering solidarity and mutual understanding, networking, and creating opportunities for women's empowerment. Self-help groups also have the potential to reduce disparities between marginalised and not so marginalised communities (38, 39).

Self-help groups complement digital health and self-care interventions to improve access to MH services. For example, a study in a hospital in northern India included general details about pregnancy, self-care preventive practices, and self-management of minor illnesses during pregnancy either through a mobile app or WhatsApp messages compared to a comparison group who received standard care. Pregnant women who received the mHealth education intervention showed significantly improved knowledge, attitudes, and practices around pregnancy than the comparison group (40).

## Discussion

### Uptake of pro-poor MH policies, women's access and self-care

The several national pro-poor MH policies described above launched between 2005 and 2019 have improved access to MH services for millions of women in India over the last two decades. Although there has been an increase in women using the schemes, particularly in the uptake of ANC checkups and institutional deliveries, studies evaluating the impact (of PMSMA) on the improvement in ANC quality and coverage are scarce. Nevertheless, pregnant women value CT (such as in JSY) and the perceived health benefits highlighting the importance of informing them about the MH services and providing quality care (41). However, JSY evaluations suggest that financial incentives may be an imprecise tool for changing health-related behaviours, and therefore, financial incentives must be used cautiously (42).

Additionally there has been mixed uptake across the board including inadequate penetration to hard-to-reach areas, high implementation and infrastructure costs, poor sustainability and scalability, limited adaptability to local needs and women continue to incur numerous OOPE across the MH journey for facility-based services While financial incentives have the potential to increase demand for and improve access to quality MH services and reduce catastrophic expenditure (4, 43–47), evidence suggests that some MH policies are underfunded (such as PMMVY), the administrative enrolment procedures are onerous, and the process of accessing benefits is slow and burdensome as the policies may be underfunded at times. One unexpected outcome of JSY was that it drew women to the public sector, resulting in overcrowding and a perceived poorer quality of obstetric care in public facilities (6) and less able to treat emergency obstetric complications. This resulted in women and their families approaching private facilities for treatment (4), in such situations, leading to greater costs. A study on PM-JAY's effect on financial risk protection of pregnant mothers, did not affect distress financing for delivery in private healthcare facilities but elsewhere PM-JAY enrolment was associated with a decrease in OOPE due to private hospitalization (5).

There continues to be reports of lack of information that women, and their families have about pro-poor MH policies in India therefore reducing uptake (8, 11, 16, 18, 48). These gaps include poor awareness of PM-JAY insurance scheme, and free entitlements of JSSK as well as a lack of knowledge about JSY, such as access to free normal delivery, medicines, and consumables, and free caesarean section and blood transfusion (if required) (6). Moreover some women reported that the various requirements—such as needing a bank account—to receive CT funds prevented them from accessing the system.

The Government of India has classified nine large northern and northeastern states as high-focus states, including Assam (49). The facility delivery rate has increased since the inception of demand-side financing and pro-poor strategies have played an important role in reducing financial barriers to access to MH services. However, these nine states continue to have higher number of maternal deaths than the national average (2, 50). Wealth-based inequalities decreased significantly at the national level for institutional deliveries, but there were varied results in low-performing states for ANC and PNC uptake, suggesting a need for greater focus on continuity of care throughout the MH journey (8). While there have been efforts to improve quality, respectful maternity care through the LaQshya and Surakshit Matritva Aashwasan programmes (launched in 2017 and 2019 respectively), policies that aim to improve women's health services need to also consider maternal autonomy and empowerment (51). Increased targeting of hard-to-reach populations and strengthening public health programs linked to MH care is critical for Universal Health Coverage. Providing more information for women to make decisions and better linkages between public and private facilities and raising awareness on services available including to those living in remote areas are required. Creating stronger linkages and interoperable systems between schemes may improve the enrolment and disbursement processes for women, which is what the NHPM aims to do. Digital interventions can drive women's autonomy and improvement by linking health programs to digital health interventions to amplify access to services, reach different population segments, and reduce OOPE.

### How digital interventions can support women's self-care and empowerment

One intervention currently underway in Assam is testing a chat-based digital support system promoting women's autonomy over their health-seeking behaviours, strengthening self-care, and facilitating access to quality MH services. The solution equips pregnant women with essential information and access to both private and public sector health providers. It makes the connections that they need for a safe and respectful MH journey (52). The e-SAATHI (***S****trengthening **A**NC/PNC* via ***A****skNivi **T**ailored **H**ealth **I**nformation, referrals, and follow-up*) intervention is powered by askNivi. It empowers pregnant women with timely and gestationally staged information, promotes self-care, and provides actionable referrals during pregnancy, childbirth and up to 15 weeks postnatal. Pregnant/postpartum women receive 2–3 tailored messages per week, including information on the financial incentives described here, referrals when needed and opportunities to provide feedback on care received. The solution provides access to quality MH services from a pregnant woman's perspective by adapting and scaling a woman-centred chat-based health navigation system, linking them to affordable and quality private and public MH providers in three of the ten districts of Assam (over 170 facilities by mid-2024). The aim is to make the connections needed for a safe and respectful MH journey. Developed using national and international MH guidelines, the digital intervention is culturally appropriate and sensitive to local contexts by pre-engagement with local women and MH providers, including members of the state ObGyn Society. Adding ASHAs' presence alongside the chatbot to reinforce information on the importance of health and the uptake of public healthcare, including the Government of India's pro-poor MH policies, can only enhance women's agency, timely care-seeking, especially for those with less access to mobile phones and in remote areas (11). The e-SAATHI intervention has the potential to both provide an interface with and leverage for multiple future interventions linked to the health system and ABDM.

## Conclusion

Collaboration beyond the health sector is a critical success factor in reducing maternal complications and deaths especially in the nine northern and northeastern states with the highest maternal mortality. The pro-poor maternal health policies introduced by the Government of India over the last two decades have gone some way to increase women's access to services. Combined with a renewed emphasis on women's self-care interventions for MH, mobile health ecosystems and networks can support women with information on accessing cash transfers for childbirth, gestation appropriate health messages, self-efficacy, and autonomy. The intersection of these three operational spaces—pro-poor policies, self-care and digital interventions—provide opportunities to expand women's agency, knowledge, self-care behaviours, and access to quality facility-based MH care for a positive pregnancy and postnatal experience. Future work on how to expand such a comprehensive approach is more likely to be effective if the programme accounts for multiple contextual factors, including understanding the nature and success of national pro-poor MH policies in each state, how the public and private health systems function and interact, local health facility dynamics, social determinants of health as well as engaging women in the process to improve maternal and newborn health outcomes.

## Data Availability

The original contributions presented in the study are included in the article/Supplementary Material, further inquiries can be directed to the corresponding author.
